# Advancing Pancreatic Cancer Prediction with a Next Visit Token Prediction Head on Top of Med-BERT

**DOI:** 10.3390/cancers17030516

**Published:** 2025-02-04

**Authors:** Jianping He, Laila Rasmy, Degui Zhi, Cui Tao

**Affiliations:** 1 McWilliams School of Biomedical Informatics, UTHealth at Houston, Houston, TX 77030, USA; jianping.he@uth.tmc.edu (J.H.); laila.rasmy.gindybekhet@uth.tmc.edu (L.R.); 2 Department of Artificial Intelligence and Informatics, Mayo Clinic, Jacksonville, FL 32224, USA

**Keywords:** foundation model, pancreatic cancer, masked language model

## Abstract

Background: Electronic Health Records (EHRs) encompass valuable data essential for disease prediction. The application of artificial intelligence (AI), particularly deep learning, significantly enhances disease prediction by analyzing extensive EHR datasets to identify hidden patterns, facilitating early detection. Recently, numerous foundation models pretrained on extensive data have demonstrated efficacy in disease prediction using EHRs. However, there remains some unanswered questions on how to best utilize such models, especially with very small fine-tuning cohorts. Methods: We utilized Med-BERT, an EHR-specific foundation model, and reformulated the disease binary prediction task into a token prediction task and a next visit mask token prediction task to align with Med-BERT’s pretraining task format in order to improve the accuracy of pancreatic cancer (PaCa) prediction in both few-shot and fully supervised settings. Results: The reformulation of the task into a token prediction task, referred to as Med-BERT-Sum, demonstrated slightly superior performance in both few-shot scenarios and larger data samples. Furthermore, reformulating the prediction task as a Next Visit Mask Token Prediction task (Med-BERT-Mask) significantly outperformed the conventional Binary Classification (BC) prediction task (Med-BERT-BC) by 3% to 7% in few-shot scenarios with data sizes ranging from 10 to 500 samples. These findings highlight that aligning the downstream task with Med-BERT’s pretraining objectives substantially enhances the model’s predictive capabilities, thereby improving its effectiveness in predicting both rare and common diseases. Conclusions: Reformatting disease prediction tasks to align with the pretraining of foundation models enhances prediction accuracy, leading to earlier detection and timely intervention. This approach improves treatment effectiveness, survival rates, and overall patient outcomes for PaCa and potentially other cancers.

## Simple Summary

Pancreatic cancer (PaCa) is estimated to be the fourth leading cause of cancer death in men, following lung, colon, and prostate cancers, and the third leading cause in women, following lung and breast cancers. PaCa is often referred to as a “silent killer” because symptoms typically manifest only in the late stages of the disease. Consequently, early detection is crucial for improving patient outcomes. This study explores the use of electronic health records (EHRs) to enhance the prediction of PaCa onset. Our research leverages Med-BERT, a foundation model designed for EHR data, to improve early detection using deep learning techniques. By aligning the prediction task with Med-BERT’s pretraining task, we aimed to enhance its accuracy, especially in scenarios with limited data. This approach can facilitate the earlier detection of PaCa in patients, thereby improving their prognosis.

## 1. Introduction

Pancreatic cancer (PaCa) is estimated to be the fourth leading cause of cancer deaths in men, following lung, colon, and prostate cancers, and the third leading cause in women, following lung and breast cancers [1]. Despite significant advancements over the past two decades aimed at improving clinical outcomes, the overall 5-year survival rate for PaCa remains just 11%, with surgical resection being the only viable treatment option [2]. Patients diagnosed in the very early stages of PaCa have the potential for a cure through a combination of surgery, immunotherapy, chemotherapy, and radiotherapy. However, PaCa is characterized by a subtle onset, a lack of distinct symptoms, its specific anatomical location, a low success rate of surgical resection, and a high recurrence rate [2]. These factors collectively render early diagnosis highly challenging, with the disease typically being identified at advanced stages [3]. Therefore, it would be significant if PaCa could be detected at the early disease stage to improve patient prognosis and increase the chances of successful treatment.

Electronic health records (EHRs) data contains a large amount of potentially valuable information for medical research and diagnosis [4]. EHR-based disease prediction can be a very significant aspect of healthcare as it allows for the early detection of illnesses [5]. In the context of PaCa, emerging studies have focused on predicting disease risk using real-world clinical records from large patient cohorts. These studies aim to facilitate the design of cost-effective surveillance programs for early detection [6,7].

Artificial intelligence (AI) techniques, including machine learning and other computational methods, are increasingly used in PaCa prediction to analyze large amounts of heterogeneous EHR data [3,6,7,8,9,10,11]. In recent years, transformer-based models have demonstrated significant potential in PaCa prediction. These models can achieve exceptionally high-performance levels when provided with sufficient high-quality training data [3,6,7,8,9,10,11]. The necessity of large, annotated datasets is particularly critical for transformer-based models, as they require extensive data to learn complex representations of the input domain. In the absence of enough and adequate training data, these models may experience underfitting, leading to suboptimal performance on new, unseen data [12]. Therefore, developing transformer-based models that can effectively perform PaCa prediction in few-shot scenarios is of paramount importance.

A foundation model is a large-scale deep learning model trained on extensive, diverse datasets, capable of performing a wide range of tasks across different domains [13]. These models are highly adaptable, allowing for customization to specific applications without the need for developing new AI systems from scratch. Foundation models serve as a versatile base for creating more specialized models efficiently and cost-effectively, exemplified by AI technologies like BERT, GPT, and others that can understand language, generate text and images, and engage in natural conversations [13]. There are a few specialized foundation models available using structured EHR data and it is crucial to optimize their application to achieve better outcomes.

Med-BERT is an encoder-based EHR foundation model, i.e., a pre-trained contextualized embedding model specific to disease prediction, which is trained on structured diagnosis codes, medication codes, and procedure codes [5,14]. Although Med-BERT has shown promising performance in disease prediction studies, including PaCa prediction, there is still potential for further improvement, especially in few-shot settings [5,14].

We hypothesize that reformulating the downstream task of predicting the onset of PaCa to align with the formulation of the token prediction used for Med-BERT pre-training could enhance its performance, particularly in the few-shot scenario. Our results indicate that this reformulation enhances Med-BERT’s performance in nearly all settings, with more pronounced improvements observed in few-shot scenarios.

## 2. Related Work

Numerous BERT variants, such as BioBERT [15] and ClinicalBERT [16], have been pretrained on clinical texts and are utilized for various clinical NLP tasks. These variations inherit the BERT architecture and pretraining tasks, specifically employing the Masked language Model (MLM) objective during the pretraining stage to train most transformer-based language models. Prompt tuning incorporates a prompt template—a text segment with mask tokens—into the initial input, transforming various downstream NLP tasks into MLM formats. This adjustment aligns target tasks with the pre-training structure, enhancing compatibility with the foundation models and learned attentions within all BERT variants [17].

Studies have shown promising results for prompt tuning in the clinical NLP field. For instance, Peng et al. [18] and He et al. [19] demonstrated its effectiveness in named entity and relation extraction. Taylor et al. [20] successfully applied prompt tuning to several clinical tasks, including ICD-9, ICD-9 Triage Task, In-Hospital Mortality, and Length of Stay in the ICU. Zhang et al. [21] utilized prompt tuning for the classification of clinical notes, while Maharjan et al. [22] applied it to clinical question answering. In all the above studies, the success of the research relies on text-based input for foundation models, where the prediction tasks are converted to a MLM format.

Given the limited availability of BERT-based foundation models trained on structured EHR data, Med-BERT stands out as a model trained on the largest patient cohort, with the full trajectory of over 20 million patients’ data [5]. Med-BERT leveraged the original BERT framework, including its architecture and training methodology, and pretrained on structured EHR data. It employed the MLM as one of the pretraining tasks, directly derived from the original BERT paper. This task involved predicting the presence of any code based on its context, with an 80% probability of replacing a code with [MASK], a 10% probability of replacing it with a random code, and a 10% probability of leaving it unchanged. This task is central for the training of any encoder-only based model [3].

Therefore, we hypothesized that if the task of PaCa is reformulated similarly to the token prediction task, it could lead to improved performance. Therefore, we reformulated the PaCa Binary Classification Prediction Task into a Token Prediction Task and a Next Visit Mask Token Prediction Task. A Token Prediction Task transforms the main outcome objective from a simple binary label for PaCa (yes/no) into the probability of the patient getting any PaCa relevant ICD-10 codes in a later visit, being a part of the vocabulary that Med-BERT was pre-trained on. The Next Visit Mask Token Prediction Task extends this approach by adding the Masked token to the first future (next) visit, so that the downstream task formulation will be shifted from a simple classification task into an MLM task. To our knowledge, no prior studies have attempted to reformulate the disease prediction downstream task in this way to improve the EHR-based foundation models’ performance.

## 3. Materials and Methods

### 3.1. Materials

To evaluate the added value of reformulating the disease prediction downstream task into a pretraining format to improve prediction accuracy, we utilized the same PaCa cohort used in the Med-BERT v2 study [14]. This cohort consists of 31,243 patients, including 12,273 cases and 18,970 controls. The dataset was split into training, validation, and test sets in a ratio of 7:1:2, resulting in up to 21,871 patients for fine-tuning, 3124 patients for validation, and 6248 patients for testing.

### 3.2. Methods

#### 3.2.1. Reformulating PaCa Binary Classification Prediction Task into Token Prediction Task and Next Visit Mask Token Prediction Task

**Figure 1:**
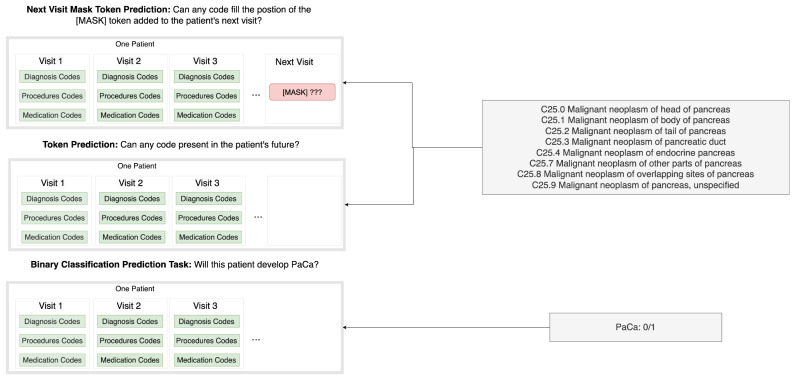
The Principle of Reformulating the PaCa Binary Classification Prediction Task into a Token Prediction Task and a Next Visit Mask Token Prediction Task.

As illustrated in Figure 1, visits 1, 2, up to visit i correspond to the patient’s diagnosis, medications, and procedures until the current visit i. Commonly, the PaCa risk prediction task is approached as a binary classification problem, which we refer to as Binary Classification Prediction (Med-BERT-BC). In contrast, our proposed approach is to estimate the probability of a PaCa ICD-10 code occurring in the future (i + 1), referred to as Token Prediction (Med-BERT-Sum), or to estimate the probability of a PaCa ICD-10 code filling the position of a MASK token added to the next visit (i + 1), which we refer to as Next Visit Mask Token Prediction (Med-BERT-Mask).

The main modification in the prediction task reformulation is the design of the outcome label. So instead of predicting directly if the patient will develop PaCa in the future (simple binary label: yes/no), we will predict if the patient will have any of the eight tokens representing the following ICD-10 codes for PaCa in any future visit, namely {C25.0 Malignant neoplasm of head of pancreas; C25.1 Malignant neoplasm of body of pancreas; C25.2 Malignant neoplasm of tail of pancreas; C25.3 Malignant neoplasm of pancreatic duct; C25.4 Malignant neoplasm of endocrine pancreas; C25.7 Malignant neoplasm of other parts of pancreas; C25.8 Malignant neoplasm of overlapping sites of pancreas; C25.9 Malignant neoplasm of pancreas, unspecified}. This modification transforms the predicted label to utilize a subset of the (tokens) labels used during Med-BERT pretraining phase, which we refer to as the PaCa Label.

For the Binary Classification Prediction task, the full patient context vector is used as input to a linear neural layer, implemented as the binary classification head. Med-BERT-Sum utilizes the full patient context vector in the same manner as is used for the binary classification model (MedBERT-BC). However, the dot product of the full patient context vector and the PaCa ICD-10 token embedding tensors (PaCa token based label) are computed to estimate the probability of each token representing the patient’s future. For MedBERT-Mask, a [MASK] token is added to the input sequence at the position corresponding to the next future visit (i + 1), which is used for the visit embedding layer of Med-BERT. The dot product of the contextual vector at the [MASK] token position and the PaCa label tensor—including the corresponding token embeddings of the PaCa Label—is computed to estimate the probability of each token filling the [MASK] position.

It is worth noting that, unlike the Med-BERT MLM pretraining task, we only used the token embedding for the above PaCa ICD-10 codes to calculate only the probabilities of these tokens instead of the full tokens list in the Med-BERT vocabulary given our specific PaCa prediction use case, and accordingly, our objective here was slightly modified to determine if any of the PaCa corresponding ICD-10 tokens have a high probability rather than finding the code with the highest chance of being the best fit, which was used for the more generalizable pretraining objective.

Therefore, for Med-BERT-Sum and Med-BERT-Mask, our intermediate output is a vector whose length matches the number of PaCa ICD-10 codes (sub-labels), eight in our case. Each element in this vector indicates the probability of this token to present in the next visit. We then use the maximum probability from this intermediate output for the final PaCa prediction evaluation.

#### 3.2.2. The Scheme of Utilizing Med-BERT for PaCa Onset Prediction

**Figure 2:**
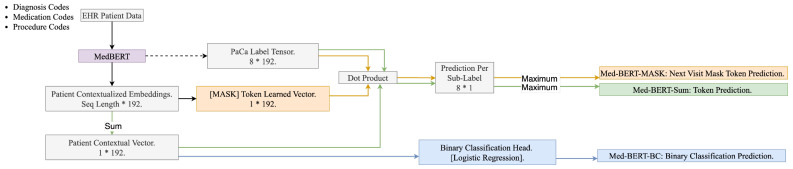
The Scheme of Utilizing Med-BERT for PaCa Onset Prediction. (we use x * y to represent the dimension of the tensors).

Figure 2 illustrates the scheme of utilizing Med-BERT for PaCa onset prediction. As illustrated in Figure 2, we used the full patient trajectory before the index date (visit i) in a sequential format where clinical codes including diagnosis, medication, and procedures are sorted by the event date and time within visits’ sequences. We first mapped the clinical codes to the Med-BERT tokens. Then, we projected the data to the Med-BERT v2 model. Within Med-BERT, the tokens get projected to the token embedding layer and the visits sequence to the visit embedding layer to generate the static patient representation, which is then projected to the transformer layers to get the contextualized embeddings for each patient.

In the original PaCa Binary Classification Prediction task (Med-BERT-BC), we initially derived patient contextualized representations with dimensions of [sequence length, embedding dimension]. Subsequently, by summing the values of the contextualized embeddings along the sequence length dimension, we generated a patient contextual vector with dimensions of [1, embedding dimension]. This patient contextual vector was then employed as input for the binary classification head to generate the final prediction. Additionally, the same patient contextual vector was employed in the token prediction head (MedBERT-Sum), as described in Section 3.2.1, to perform the PaCa onset prediction task.

By reformulating the PaCa Binary Classification Prediction Task into a Token Prediction Task or a Next Visit Mask Token Prediction Task, our objective was to identify the most probable token from the subset of the target concept ‘PaCa’ that can present in a patient’s future or replace the [MASK] token in next visit. To achieve this, we extracted the PaCa Label Tensor from the Med-BERT token embedding layer, with a dimension of [8, embedding dimension].

For Med-BERT-Sum, we multiplied the patient contextual vector by the PaCa Label Tensor, resulting in a vector whose length corresponds to the number of PaCa labels. The highest score within this vector was selected to determine if a PaCa-related label could be assigned to a future visit. For Med-BERT-Mask, the masked token learned vector was multiplied by the PaCa Label Tensor, similarly resulting in a vector whose length corresponds to the number of PaCa labels. Again, the highest score within this vector was used to infer if a PaCa-related label could be assigned to the masked token.

Unlike the traditional binary classification, which produces a single probability indicating whether a patient will develop PaCa, our Med-BERT-Sum and Med-BERT-Mask models generate eight probabilities, each corresponding to the likelihood of a specific PaCa-related label appearing in the patient’s future visits or replacing the [MASK] token in the next visit. The highest probability among these is used to determine whether the patient is likely to develop PaCa. If the maximum probability is close to zero, it indicates that the patient is unlikely to develop PaCa, similar to the outcome in binary classification. As a result, non-PaCa ICD codes were excluded from the study, as they are irrelevant to the task. In Figure 2, the blue path represents the binary prediction, Med-BERT-BC; the green path signifies the token prediction, Med-BERT-Sum; and the orange path denotes the next visit mask token prediction, Med-BERT-Mask.

#### 3.2.3. Baseline Models

In our study, the baseline models employed included Logistic Regression (LR), Long Short-Term Memory (LSTM), Bidirectional LSTM (BiLSTM), Gated Recurrent Unit (GRU), and Bidirectional GRU (BiGRU). These models were chosen due to their proven effectiveness in handling sequential data, allowing us to benchmark our proposed approach against well-established architectures in the field [5].

#### 3.2.4. Experimental Settings

We evaluated the model’s performance using the Area Under the Receiver Operating Characteristic Curve (AUROC). The sequence length was fixed at 64, truncating any sequences that exceeded this length. Clinical diagnoses, procedures, and medications for each patient were arranged in reverse chronological order. By setting the sequence length to 64, we retained the most recent 64 codes up to the patient’s last visit. The batch size was configured to 100. For the learning rate, we employed a value of 0.001 for the Med-BERT models, GRU, and LSTM, while a learning rate of 1 × 10^−5^ was used for BiGRU and BiLSTM. We assessed the performance of all models in both few-shot scenarios and fully supervised settings. The data sample sizes varied [10, 20, 30, 40, 50, 100, 200, 300, 400, 500, 1000, 2000, 3000, 4000, 5000, 10,000], as did the full dataset. For data sizes of 10, 20, 30, 40, 50, 100, 200, 300, 400, 500, and 1000, we used an equal split of positive and negative instances for both the training and validation datasets. For example, with a data size of 10, we utilized 5 positive instances and 5 negative instances for both fine-tuning and validation. For data sizes of 2000, 3000, 4000, 5000, and 10,000, we employed the entire validation dataset for validation, while maintaining an equal split of positive and negative instances for the fine-tuning dataset. For the full data size, the entire fine-tuning and validation datasets were used. Regardless of the experimental data size, the complete test dataset was used for testing. For each data size, we conducted 3 runs to calculate the mean and standard deviation of the AUROC.

#### 3.2.5. External Validation

For external validation, we utilized the publicly available EHRSHOT dataset [23], a smaller cohort comprising 3,810 patients, including 199 cases and 3,611 controls. The dataset was divided into training, validation, and test sets using a 7:1:2 ratio, resulting in up to 2,667 patients for fine-tuning, 381 patients for validation, and 762 patients for testing. The experimental settings for this cohort closely align with those employed in the Med-BERT-v2 study cohort. However, a minor difference exists in the data sample size configuration due to the limited size of cases in the EHRSHOT dataset, which consists of only 199 cases. The data sample sizes considered in this cohort and the full dataset were [10, 20, 30, 40, 50, 100, 200, 300, 400, 500, 1000]. For sizes 10–30, training and validation datasets had equal positive and negative samples. For sizes 40–200, training included equal positive and negative samples, while validation used all positive instances and sampled negatives to match the target size. For size 300, both training and validation used all positive instances and sampled negatives to match the target size. For sizes 400–1000, training included all positives and sampled negatives, while validation used the full dataset. For the full size, all training and validation data were used.

## 4. Results

**Figure 3:**
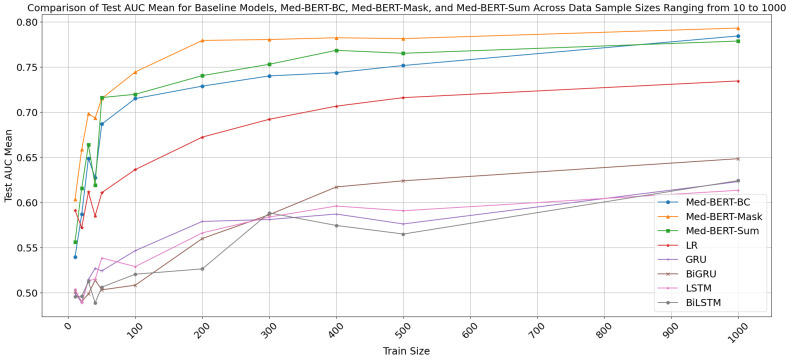
Comparison of Test AUC Means for Baseline Models, Med-BERT-BC, Med-BERT-Mask, and Med-BERT-Sum Across Data Sample Sizes Ranging from 10 to 1000.

**Figure 4:**
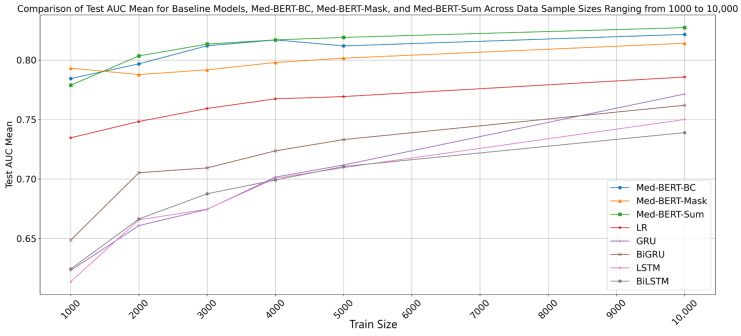
Comparison of Test AUC Means for Baseline Models, Med-BERT-BC, Med-BERT-Mask, and Med-BERT-Sum Across Data Sample Sizes Ranging from 1000 to 10,000.

Figure 3 and Figure 4 illustrate the AUC means on the test set for Baseline Models, Med-BERT-BC, Med-BERT-Mask, and Med-BERT-Sum across data sample sizes ranging from 10 to 1000, and 1000 to 10,000, respectively. The x-axis represents the training size, while the y-axis shows the AUC means on the test set. From Figure 3 and Figure 4, it is evident that all models exhibit an increase in performance as the fine-tuning data size grows, indicating improved performance with more data. Notably, Med-BERT models, including Med-BERT-BC, Med-BERT-Sum and Med-BERT-Mask, consistently achieve significantly higher AUC scores compared to the baseline models, highlighting their superior performance.

As illustrated in Figure 3 and Figure 4, Med-BERT-Sum demonstrates a modest improvement in average AUC performance compared to Med-BERT-BC across almost all data sizes, highlighting the effectiveness of leveraging Med-BERT pretraining vocabulary token embeddings for the PaCa label tensor. A similar trend was observed in the EHRSHOT validation set (Supplementary Figure S1). Furthermore, when compared to Med-BERT-BC, Med-BERT-Mask exhibited a marked increase in performance, with average AUC improvements ranging from 3% to 7% in few-shot scenarios with data sample sizes from 10 to 500 (Supplementary Table S1). This suggests that reformulating the prediction task into a Pretraining format—Mask Token Prediction task enhances performance in few-shot settings. Similarly, within the EHRSHOT validation set, Med-BERT-Mask also outperforms Med-BERT-BC in few-shot scenarios with balanced sets (Supplementary Figure S1).

As the training size increases, the performance of all three Med-BERT models stabilizes. When the data size is below 1000, Med-BERT-Mask continues to outperform the other models, achieving approximately 0.79 AUC with 1000 samples, which is 1% higher than Med-BERT-BC’s 0.78 AUC. When the data size is larger than 1000, Med-BERT-BC and Med-BERT-Sum both exhibit performance improvements with increasing training sizes, with Med-BERT-BC reaching an AUC of approximately 0.82 at 10,000 samples. Med-BERT-Mask initially shows a decline in performance after 1000 samples, followed by a gradual improvement, but does not surpass the performance of Med-BERT-BC and Med-BERT-Sum beyond 1000 samples. Med-BERT-Sum achieves the highest AUC of approximately 0.83 with 10,000 training samples, consistently outperforming Med-BERT-Mask once the fine-tuning sample size exceeds 1000. Additionally, Med-BERT-Sum slightly surpasses Med-BERT-BC as the training size increases, and consistently demonstrates superior performance compared to Med-BERT-BC across training sizes ranging from 2000 to the full fine-tuning set of 21,871 samples. These observations suggest that with sufficient training data, Med-BERT-BC performs well, while utilizing Med-BERT pretraining vocabulary token embeddings for the PaCa label tensor is advantageous in both few-shot scenarios and larger training cohorts.

The observed sharp decrease in the AUC mean for training sizes between 40 and 50, as shown in Figure 3, may be attributed to the presence of outliers and the inherent variability in smaller datasets. To further validate the model’s robustness, we conducted additional repeated experiments on smaller datasets with training sizes of less than 100, performing 10 runs to assess the consistency of the results. These findings are presented in Supplementary Figure S2. The results indicate that while outliers can occasionally impact performance in smaller datasets, the overall trend remains consistent. Notably, Med-BERT-Mask demonstrates robust performance with limited data, reinforcing the model’s effectiveness under these conditions.

**Figure 5:**
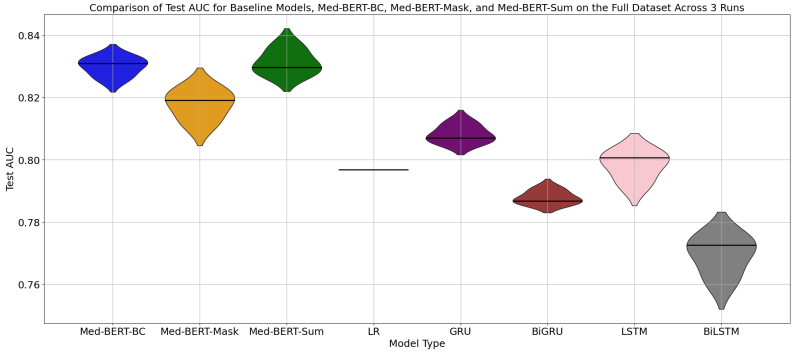
Comparison of Test AUCs for Baseline Models, Med-BERT-BC, Med-BERT-Mask, and Med-BERT-Sum on the Full Dataset Across Three Runs.

Figure 5 is a violin plot comparing the Test AUC for eight different models: Med-BERT-BC, Med-BERT-Mask, Med-BERT-Sum, LR, GRU, BiGRU, LSTM and BiLSTM across three runs using the full dataset. The x-axis represents the model type, and the y-axis shows the test AUC scores. Each “violin” represents the distribution of the test AUC values for one model type. The solid lines within each plot indicate the median. We can see that with fully supervised settings, Med-BERT models generally achieve better performance than baseline models.

Med-BERT-BC has a relatively narrow distribution, indicating a consistent performance across the three runs. Med-BERT-Mask has a wider distribution, indicating more variability in performance. The test AUC values for this model are spread out more compared to Med-BERT-BC. Similar to Med-BERT-Mask, Med-BERT-Sum also shows a wider distribution of test AUC values, indicating variability in the results. While Med-BERT-Sum’s results lack stability, it demonstrates the potential to achieve higher performance in certain runs.

Supplementary Table S1 presents the detailed results including the AUC mean, standard deviation, and performance boost compared to Med-BERT-BC on the test set for all models across all data sample sizes. The results indicate that Med-BERT-Mask outperforms Med-BERT-BC when trained on smaller datasets, highlighting the advantage of reformulating the downstream task into the Mask Token Prediction task in few-shot scenarios. Additionally, Med-BERT-Sum demonstrates a slight performance improvement over Med-BERT-BC across both few-shot and larger dataset settings. These findings suggest that reformulating the PaCa label tensor to utilize Med-BERT’s pretraining vocabulary token embeddings consistently enhances model performance. In contrast, the MLM task formulation proves particularly beneficial in few-shot settings, providing substantial performance gains under these conditions.

## 5. Discussion

Med-BERT-BC utilizes binary classification directly on the PaCa prediction task. Med-BERT-Sum uses the Med-BERT pretraining vocabulary token embeddings for the PaCa label tensor, which is its primary distinction from Med-BERT-BC. Med-BERT-Sum demonstrated slightly superior performance compared to Med-BERT-BC across most data sizes (ranging from 10 to a fully supervised setting), suggesting that utilizing the Med-BERT pre-trained vocabulary token embeddings for the PaCa label tensor is effective for boosting the fine-tuned model performance. Med-BERT-Mask not only incorporates the Med-BERT pre-trained vocabulary token embeddings but also reformulates the binary classification task into an MLM task, aligning with Med-BERT’s pretraining objective. The observation that Med-BERT-Mask achieves higher AUC scores compared to Med-BERT-BC with limited fine-tuning data underscores the efficacy of reformulating the PaCa prediction downstream task into a MLM framework for few-shot scenarios as hypothesized. These findings collectively demonstrate that reformulating the PaCa prediction task into the formulation of the token prediction used for Med-BERT pre-training could enhance its predictive capabilities for PaCa even with the availability of a few tens of cases data and equal numbers of controls. So, for example, with 100 PaCa cases and 100 controls, we can fine-tune Med-BERT-Mask for PaCa prediction and achieve a prediction accuracy of around 78%, which is similar to the AUC that can be achieved after fine-tuning a binary classification head on top of Med-BERT for 1000 samples. Consequently, this study encourages us to formulate disease prediction downstream tasks in a manner akin to the pretraining task format when utilizing foundation models like Med-BERT in order to maximize their performance in few-shot settings. The external validation further corroborated these trends. Specifically, Med-BERT-Sum demonstrated superior performance, consistently outperforming Med-BERT-BC across all dataset sizes. Notably, Med-BERT-Mask achieved performance levels comparable to Med-BERT-Sum, particularly in the context of few-shot balanced datasets (less than 300 samples in the EHRSHOT cohort).

Med-BERT-Sum slightly outperformed Med-BERT-BC across all the data sizes, demonstrating the added value of using the pretraining vocabulary tokens as an intermediate label for prediction. Med-BERT-Mask excels in few-shot scenarios due to its ability to effectively leverage pre-trained knowledge through task reformulation. This approach optimally utilizes the generalized patterns and representations from the pre-trained foundation model, enhancing performance with limited fine-tuning data. However, as the fine-tuning dataset size increases, the classification head can better converge in Med-BERT-BC, which allows the Med-BERT to learn more discriminative features tailored to the specific task of predicting PaCa onset. Therefore, the extensive task-specific data reduces the need for reformulation, as direct optimization yields better and more stable results.

In the domain of clinical decision support, firstly, our method enhances the accuracy of PaCa prediction. By offering more reliable predictions, our approach can aid healthcare professionals in making better-informed decisions, ultimately leading to improved patient outcomes. This provides substantial benefits for the clinical field, including PaCa, other cancers, and even non-cancerous diseases. Furthermore, the challenge of diagnosing rare diseases is exacerbated by the limited datasets available for training models to achieve high performance with new patients. Our method’s strong performance in few-shot scenarios demonstrates the feasibility of AI-based clinical decision support systems for rare diseases. This success addresses the unique challenge posed by infrequent diseases and encourages further innovation in AI-driven healthcare, potentially extending benefits to a broader range of medical applications.

If the clinical workflow incorporates BERT-based models like Med-BERT, our approach suggests reformulating the downstream task into a token prediction task (Med-BERT-Sum) or a masked token prediction task (Med-BERT-Mask) based on the availability of training data. Med-BERT-Mask is advantageous in scenarios with limited data, while Med-BERT-Sum performs better with larger datasets. If clinical workflows employ models that are not BERT-based, the downstream task can still be tailored to fit the pretraining task format of the specific model in use. This strategy involves analyzing the pretraining objective of the foundation model and reformulating the clinical prediction task to resemble this objective, which can significantly enhance the model’s performance. However, we acknowledge the challenges in implementing this approach, such as the need for expertise in understanding and adapting the task formulations.

Our method presents several limitations that warrant further investigation in future research. First, this study utilized the PaCa Onset Cohort, as employed in the original Med-BERT v2 study. Notably, this cohort is based on secondary clinical data, which is retrospective and generated mostly for billing purposes. Consequently, the sensitivity for PaCa within this dataset is relatively low. Furthermore, the recorded time for PaCa onset often lags behind the true diagnosis due to the inherent difficulty in diagnosing PaCa, which typically results in a billing entry only after a confirmed diagnosis. This characteristic leads to a high number of true positives, reducing the risk of false positives and contributing to an elevated positive predictive value (PPV). Regarding the control group, we undertook rigorous steps to minimize false negatives by excluding all other cancer patients, including those with benign tumors, from the cohort. Ideally, validation against manual chart reviews would enhance accuracy; however, this study prioritized algorithm development, making this limitation beyond our current scope. In future work, we plan to utilize cohorts derived from clinical data instead of billing data to mitigate these issues. Additionally, the cohort used in this study provided a relatively balanced dataset for training and evaluation purposes. However, we acknowledge that this balanced distribution does not mirror real-world conditions, where the incidence of PaCa is significantly lower in the general population. In future research, addressing class imbalance during the cohort inclusion and exclusion stages will be critical. This could involve intentionally constructing an imbalanced cohort to better represent real-world scenarios, thereby enhancing the model’s applicability in practice. Moreover, the current evaluation was restricted to the prediction of PaCa onset, which limits our understanding of the method’s generalizability to different diseases and clinical contexts. To ensure robustness and broader applicability, future studies should incorporate evaluations across a diverse array of diseases, including various cancers and non-cancerous conditions. Finally, our testing was confined to a single clinical foundation model, Med-BERT. Future evaluations should be extended to other clinical foundation models to comprehensively assess the method’s performance.

## 6. Conclusions

In this study, we reformulated the downstream task of predicting the onset of PaCa to align with the token prediction used in Med-BERT pre-training. This reformulation enhanced Med-BERT fine-tuning efficacy, resulting in superior performance compared to the conventional BC prediction design in both few-shot and fully supervised settings. Our findings highlight the potential of leveraging foundation models more effectively by aligning downstream tasks with their pretraining objectives. This strategy offers a promising avenue for developing more robust AI clinical support systems for PaCa, other cancers, and non-cancerous diseases, thereby improving patient outcomes. Future research will focus on validating these findings across diverse cancer and non-cancer contexts, as well as on different foundation models, and exploring further optimizations to enhance cancer diagnostic systems.

## Data Availability

EHRSHOT data used for external validation is available through https://som-shahlab.github.io/ehrshot-website/, accessed on 29 January 2025. Data required for Med-BERT v2 model pretraining and evaluation were used under license from data providers.

## Supplementary Materials

The following supporting information can be downloaded at: https://www.mdpi.com/article/10.3390/cancers17030516/s1, Table S1: The Test AUC Mean, Standard Deviation, and Performance Boost Compared to Med-BERT-BC for All Models across All Data Sample Sizes; Figure S1: Comparison of Test AUC Mean for Med-BERT-BC, Med-BERT-Mask, and Med-BERT-Sum on the EHRSHOT Dataset Across Data Sample Sizes Ranging from 10 to 1000; Figure S2: Comparison of Test AUC Mean for Med-BERT-BC, Med-BERT-Mask, and Med-BERT-Sum Across Data Sample Sizes Ranging from 1000 to 100 (10 runs).
