# Supplementary material for: Advancing Pancreatic Cancer Prediction with a Next Visit Token Prediction Head on Top of Med-BERT
Source: Cancers (Basel). 2025 Feb 4;17(3):516. doi: 10.3390/cancers17030516 (PMC11816036; doi:10.3390/cancers17030516)
Supplement: Supplementary file 1 [file cancers-17-00516-s001.zip › cancers-3438386-supplementary.pdf]

Table S1. The Test AUC Mean, Standard Deviation, and Performance Boost Compared to Med-BERT-BC for All Models across All Data Sample Sizes.

**Train_Size**	**Function_Type**	**Test_AUC mean (%)**	**Performance Boost (%)**	**Test_AUC std**
10	LR	59.13	5.15	0.02
10	GRU	49.95	-4.03	0.03
10	BiGRU	50.21	-3.77	0.02
10	LSTM	50.39	-3.59	0.01
10	BiLSTM	49.57	-4.41	0.02
10	Med-BERT-BC	53.98		0.04
10	Med-BERT-Sum	55.64	1.66	0.05
10	**Med-BERT-Mask**	**60.35**	**6.37**	**0.05**
20	LR	57.22	-1.47	0.07
20	GRU	48.92	-9.77	0.02
20	BiGRU	49.04	-9.65	0.01
20	LSTM	48.93	-9.76	0.02
20	BiLSTM	49.62	-9.07	0.01
20	Med-BERT-BC	58.69		0.10
20	Med-BERT-Sum	61.58	2.89	0.06
20	**Med-BERT-Mask**	**65.88**	**7.19**	**0.06**
30	LR	61.17	-3.73	0.03
30	GRU	51.48	-13.42	0.03
30	BiGRU	49.89	-15.01	0.02
30	LSTM	51.35	-13.55	0.03
30	BiLSTM	51.26	-13.64	0.01
30	Med-BERT-BC	64.90		0.04
30	Med-BERT-Sum	66.40	1.50	0.05
30	**Med-BERT-Mask**	**69.85**	**4.95**	**0.06**
40	LR	58.51	-4.25	0.04
40	GRU	52.72	-10.04	0.01
40	BiGRU	51.41	-11.35	0.00
40	LSTM	51.50	-11.26	0.01
40	BiLSTM	48.90	-13.86	0.02
40	Med-BERT-BC	62.76		0.05
40	Med-BERT-Sum	61.94	-0.82	0.03
40	**Med-BERT-Mask**	**69.35**	**6.59**	**0.04**
50	LR	61.10	-7.59	0.05
50	GRU	52.44	-16.25	0.01
50	BiGRU	50.34	-18.35	0.01
50	LSTM	53.86	-14.83	0.04
50	BiLSTM	50.64	-18.05	0.01
50	Med-BERT-BC	68.69		0.04
50	**Med-BERT-Sum**	**71.62**	**2.93**	**0.02**
50	Med-BERT-Mask	71.52	2.83	0.05
100	LR	63.65	-7.86	0.02
100	GRU	54.68	-16.83	0.03
100	BiGRU	50.86	-20.65	0.02
100	LSTM	52.91	-18.60	0.02
100	BiLSTM	52.07	-19.44	0.04
100	Med-BERT-BC	71.51		0.03

100	Med-BERT-Sum	71.99	0.48	0.02
100	**Med-BERT-Mask**	**74.46**	**2.95**	**0.01**
200	LR	67.24	-5.65	0.03
200	GRU	57.91	-14.98	0.02
200	BiGRU	56.02	-16.87	0.04
200	LSTM	56.64	-16.25	0.01
200	BiLSTM	52.66	-20.23	0.03
200	Med-BERT-BC	72.89		0.01
200	Med-BERT-Sum	74.05	1.16	0.01
200	**Med-BERT-Mask**	**77.95**	**5.06**	**0.00**
300	LR	69.22	-4.80	0.00
300	GRU	58.12	-15.90	0.02
300	BiGRU	58.65	-15.37	0.03
300	LSTM	58.40	-15.62	0.02
300	BiLSTM	58.86	-15.16	0.02
300	Med-BERT-BC	74.02		0.00
300	Med-BERT-Sum	75.30	1.28	0.01
300	**Med-BERT-Mask**	**78.05**	**4.03**	**0.01**
400	LR	70.67	-3.71	0.01
400	GRU	58.73	-15.65	0.03
400	BiGRU	61.73	-12.65	0.03
400	LSTM	59.61	-14.77	0.01
400	BiLSTM	57.47	-16.91	0.04
400	Med-BERT-BC	74.38		0.00
400	Med-BERT-Sum	76.85	2.47	0.00
400	**Med-BERT-Mask**	**78.24**	**3.86**	**0.01**
500	LR	71.61	-3.56	0.01
500	GRU	57.63	-17.54	0.02
500	BiGRU	62.41	-12.76	0.01
500	LSTM	59.09	-16.08	0.02
500	BiLSTM	56.51	-18.66	0.07
500	Med-BERT-BC	75.17		0.01
500	Med-BERT-Sum	76.52	1.35	0.01
500	**Med-BERT-Mask**	**78.15**	**2.98**	**0.01**
1000	LR	73.46	-4.99	0.00
1000	GRU	62.32	-16.13	0.01
1000	BiGRU	64.86	-13.59	0.01
1000	LSTM	61.36	-17.09	0.01
1000	BiLSTM	62.43	-16.02	0.01
1000	Med-BERT-BC	78.45		0.01
1000	Med-BERT-Sum	77.88	-0.57	0.01
1000	**Med-BERT-Mask**	**79.32**	**0.87**	**0.01**
2000	LR	74.83	-4.85	0.01
2000	GRU	66.08	-13.60	0.02
2000	BiGRU	70.53	-9.15	0.01
2000	LSTM	66.56	-13.12	0.00
2000	BiLSTM	66.64	-13.04	0.01
2000	Med-BERT-BC	79.68		0.00
2000	**Med-BERT-Sum**	**80.35**	**0.67**	**0.01**
2000	Med-BERT-Mask	78.78	-0.90	0.00
3000	LR	75.93	-5.27	0.01

3000	GRU	67.45	-13.75	0.01
3000	BiGRU	70.93	-10.27	0.00
3000	LSTM	67.46	-13.74	0.01
3000	BiLSTM	68.76	-12.44	0.01
3000	Med-BERT-BC	81.20		0.01
3000	**Med-BERT-Sum**	**81.34**	**0.14**	**0.00**
3000	Med-BERT-Mask	79.18	-2.02	0.01
4000	LR	76.74	-4.95	0.00
4000	GRU	70.16	-11.53	0.01
4000	BiGRU	72.36	-9.33	0.01
4000	LSTM	70.06	-11.63	0.01
4000	BiLSTM	69.91	-11.78	0.01
4000	**Med-BERT-BC**	**81.69**		**0.00**
4000	**Med-BERT-Sum**	**81.69**	**0.00**	**0.00**
4000	Med-BERT-Mask	79.79	-1.90	0.00
5000	LR	76.93	-4.26	0.00
5000	GRU	71.17	-10.02	0.01
5000	BiGRU	73.31	-7.88	0.01
5000	LSTM	70.95	-10.24	0.01
5000	BiLSTM	71.05	-10.14	0.01
5000	Med-BERT-BC	81.19		0.00
5000	**Med-BERT-Sum**	**81.90**	**0.71**	**0.00**
5000	Med-BERT-Mask	80.16	-1.03	0.01
10000	LR	78.57	-3.59	0.00
10000	GRU	77.15	-5.01	0.01
10000	BiGRU	76.20	-5.96	0.00
10000	LSTM	75.00	-7.16	0.01
10000	BiLSTM	73.90	-8.26	0.01
10000	Med-BERT-BC	82.16		0.00
10000	**Med-BERT-Sum**	**82.73**	**0.57**	**0.00**
10000	Med-BERT-Mask	81.41	-0.75	0.00
Full Data Size (21871)	LR	79.69	-3.31	0.00
Full Data Size (21871)	GRU	80.82	-2.18	0.00
Full Data Size (21871)	BiGRU	78.79	-4.21	0.00
Full Data Size (21871)	LSTM	79.82	-3.18	0.00
Full Data Size (21871)	BiLSTM	76.93	-6.07	0.01
Full Data Size (21871)	Med-BERT-BC	83.00		0.00
Full Data Size (21871)	**Med-BERT-Sum**	**83.13**	**0.13**	**0.00**
Full Data Size (21871)	Med-BERT-Mask	81.78	-1.22	0.00

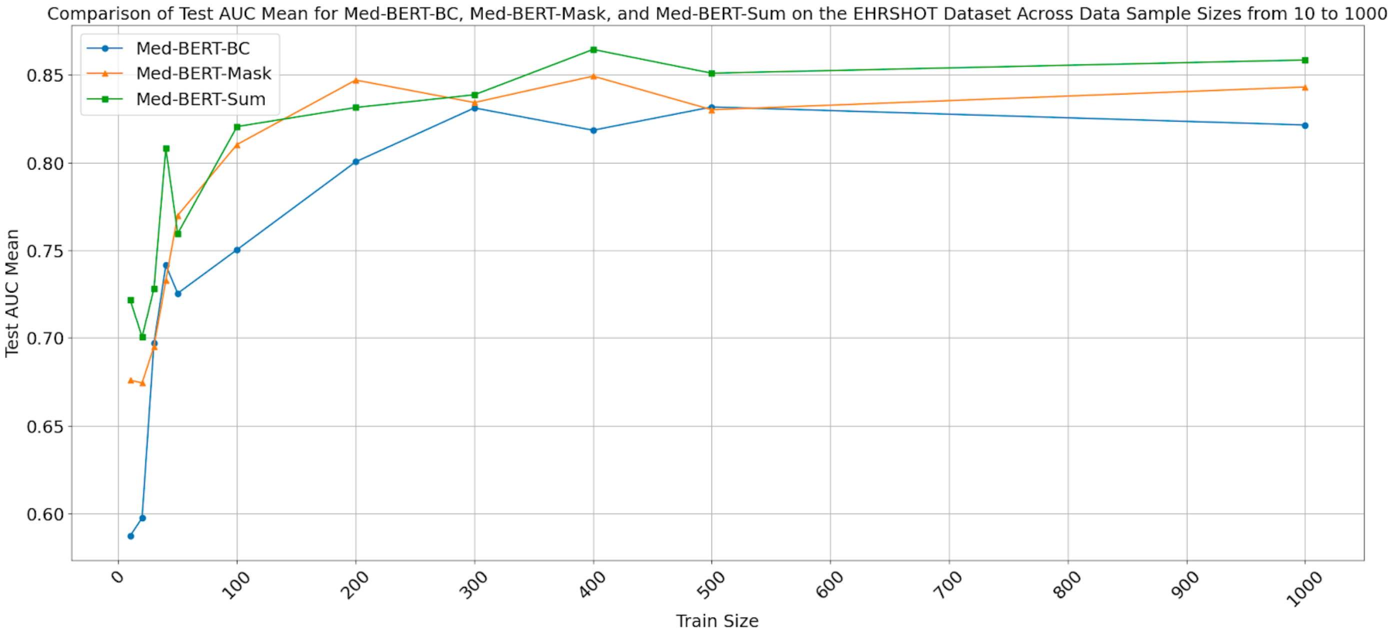

Figure S1. Comparison of Test AUC Mean for Med-BERT-BC, Med-BERT-Mask, and Med-BERT-Sum on the EHRSHOT Dataset Across Data Sample Sizes Ranging from 10 to 1000.

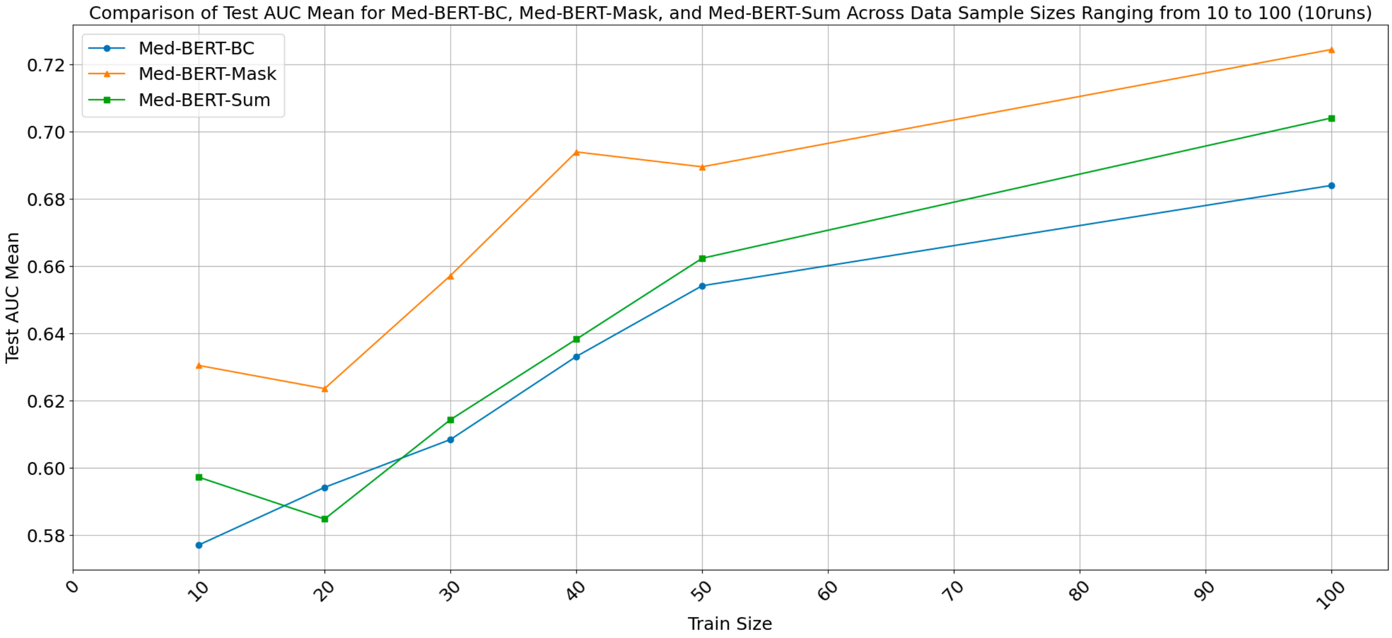

Figure S2. Comparison of Test AUC Mean for Med-BERT-BC, Med-BERT-Mask, and Med-BERT-Sum Across Data Sample Sizes Ranging from 1000 to 100 (10 runs).
